# Antibacterial interactions of pulegone and 1,8‐cineole with monolaurin ornisin against *Staphylococcus aureus*

**DOI:** 10.1002/fsn3.2870

**Published:** 2022-04-05

**Authors:** Ali Farhanghi, Javad Aliakbarlu, Hossein Tajik, Negar Mortazavi, Leila Manafi, Ghader Jalilzadeh‐Amin

**Affiliations:** ^1^ 117045 Department of Food Hygiene and Quality Control Faculty of Veterinary Medicine Urmia University Urmia Iran; ^2^ 117045 Department of Internal Medicine and Clinical Pathology Faculty of Veterinary Medicine Urmia University Urmia Iran

**Keywords:** 1,8‐cineole, combination, monolaurin, nisin, pulegone, *S. aureus*

## Abstract

The aim of this study was to investigate the antibacterial interactions of pulegone and 1,8‐cineole with monolaurin ornisin against *Staphylococcus aureus*. The individual and combined antibacterial activities of the compounds were evaluated using minimum inhibitory concentration (MIC), minimum bactericidal concentration (MBC), fractional inhibitory concentration index (FICi), and time‐kill methods. Furthermore, the mechanism of the antibacterial action of the compounds was tested by measuring the release of cell constituents. The MIC values of pulegone, 1,8‐cineole, nisin, and monolaurin were 5.85 µl/ml, 23.43 µl/ml, 6.25 µg/ml, and 0.031 mg/ml, respectively. A synergistic antibacterial activity (FICi = 0.5) was found between 1,8‐cineole and nisin. The time‐kill assay showed that the populations of *S. aureus* exposed to 1,8‐cineole, nisin, and their combination were decreased by 5.9, 5.3, and 7.1 log CFU (colony‐forming units)/mL, respectively. The combination of 1,8‐cineole and nisin also induced the highest release of cell constituents. It was concluded that the combination of 1,8‐cineole and nisin could be considered as a novel and promising combination which may reduce the required dose of each antibacterial compound.

## Graphical abstract

The antibacterial activity of pulegone and 1,8‐cineole combined with monolaurin or nisin was investigated against *Staphylococcus aureus*. A synergistic antibacterial effect was found between 1,8‐cineole and nisin. This combination also induced the highest release of cell constituents. Therefore, the combination of 1,8‐cineole and nisin could be considered as a novel and effective antibacterial combination to control *S. aureus*.

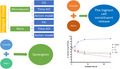

## 1 INTRODUCTION

Nowadays, the food‐borne disease has become a globally serious concern that is severely on the rise and has destructive effects on food security and human health. Based on the reports, an average of 600 million people in the world annually become infected with pathogens, or their toxins through the consumption of contaminated foods (Faour‐Klingbeil & CD Todd, 2020). *Staphylococcus aureus*, a Gram‐positive bacterium, is one of the most common food‐borne pathogens that cause food intoxication by its enterotoxins (Zhou et al., 2020). *S*. *aureus* is capable of surviving and growing in a wide range of temperatures (7–48.5°C), pH (4.0–10.0), and a high salt concentration (Guo et al., 2020). Consumers might be at risk for *S. aureus* contamination through the processing or manufacture of various foods such as ready‐to‐eat foods, cooked meat, dairy, egg, bean, aquatic products, and fresh vegetables (Ma et al., 2018).

Synthetic preservatives are used to inhibit bacterial growth and increase the shelf life and ensure food safety. Nevertheless, chemical preservatives may cause harmful effects on human health, such as headaches, palpitations, allergies, stomach cancer, asthma, skin rashes, and contact dermatitis (Sharma, 2015). In fact, due to the increasing awareness of consumers about the adverse effects of chemical antimicrobials, the tendency to use natural compounds to protect food and control food‐borne pathogens has increased significantly (Baygar, 2019).

Pulegone is a monoterpene ketone found in the leaves and flowers of several members of the mint family (Mkaddem et al., 2009). Terpenes are capable of penetrating into the bacterial cell wall, leading to denaturation of proteins and disintegration of the cell membrane, leading to cytoplasmic leakage, cell lysis, and eventually cell death (Oussalah et al., 2007). Based on the published reports, pulegone can effectively destroy *S*. *aureus*, *S*. *typhimurium*, and *Escherichia coli* (Mkaddem et al., 2009). 1,8‐cineole (eucalyptol) is a monoterpene that occurs in the essential oils of several aromatic plants and spices, including *Origanum vulgare* (oregano), *Rosmarinus officinalis* (rosemary), *Thymus vulgaris* (thyme), *Zingiber officinale* (ginger), *Coriandrum sativum* (coriander), and eucalyptus oil (Charalambous, 1994; Sebei et al., 2015). Generally, essential oils or their compounds possess the desired antibacterial effect at higher doses, but this can cause a negative sensory impact (Gutierrez et al., 2009). The combination of essential oils with other antimicrobial agents could reduce their doses and solve this problem. Some previous studies have investigated the antimicrobial interaction between 1,8‐cineol and other essential oil components such as limonene (van Vuuren & Viljoen, 2007), camphor (Viljoen et al., 2003), aromadendrene (Mulyaningsih et al., 2010), and carvacrol (De Sousa et al., 2012).

Nisin is a natural preservative used in the food industry to prevent the growth of microorganisms, especially Gram‐positive bacteria, such as *S*. *aureus* (Wang et al., 2020). However, it has been reported that some bacterial species, including *S. aureus*, may acquire resistance to nisin (Zhou et al., 2014).

Monolaurin, an ester of lauric acid, is used as an antimicrobial and emulsifier agent in the food industry. It possesses the strongest antimicrobial effect among fatty acid esters (Raeisi et al., 2016). Generally, this monoester is very effective against Gram‐positive bacteria such as *Staphylococcus* and *Streptococcus*, as well as effectively inhibits toxin production by *Staphylococci* (Lieberman et al., 2006). Higher concentrations of monolaurin may result in undesirable soapy aroma and taste (Bell & De Lacy, 1987). Antibacterial interaction between nisin and monolaurin has been previously reported (Mansour & Millière, 2001; Zhang et al., 2009).

Some previous studies have reported the individual antibacterial effect of pulegone, 1,8‐cineole, monolaurin, and nisin and their combination with other antimicrobial agents. However, there is no report on the antibacterial interactions of two essential oil components (pulegone and 1,8‐cineole) with monolaurin or nisin. Then, the objective of the current study was to evaluate the combined antibacterial activity of pulegone with monolaurin/nisin and 1,8‐cineole with monolaurin/nisin against *Staphylococcus aureus*.

## 2 MATERIALS AND METHODS

### 2.1 Materials

Brain heart infusion (BHI) broth, plate count agar (PCA), peptone water, dimethyl sulfoxide, and HCl were obtained from Merck (Darmstadt, Germany). Pulegone, 1,8‐cineole, and nisin were purchased from Sigma‐Aldrich. Monolaurin was obtained from Lauricidin Inc., Galena, IL, USA.

### 2.2 Preparation of the bacterial suspension

*Staphylococcus aureus* (PTCC1431) was procured from the microbial collection of the Department of Food Hygiene and Quality Control, Urmia University. The colonies of the bacterium grown on the PCA were transferred to BHI broth (10 ml) and incubated at 37°C for 24 h. Then, the broth containing bacteria was centrifuged at 9342 g for 10 min and washed with peptone water (0.1%) thrice. After that, 10 ml of peptone water (0.1%) was added to the bacterial pellet and resuspended. Finally, the turbidity of the bacterial suspension was adjusted to 0.1 at 600 nm (≈10^8^ CFU/ml) using a spectrophotometer (Pharmacia LKB, Uppsala, Sweden) (Mortazavi & Aliakbarlu, 2019).

### 2.3 Preparation of the antimicrobial solutions

First, 600 μl pulegone or 1,8‐cineole was mixed with 1 ml dimethyl sulfoxide (10%) to prepare their stock solutions (375 μl/ml) and 1 ml of this solution was added to 9 ml BHI broth. Then, different twofold concentrations (187.5–0.73 μl/ml) of the compounds were prepared in BHI broth from the stock solutions. To prepare the stock solution of monolaurin, 500 mg of monolaurin was dissolved in 5 ml of ethanol (96%) and 0.1 ml of this solution was added to 9.9 ml BHI broth. Then, the solution was diluted to reach concentrations ranging from 1 to 0.0156 mg/ml (Razavi‐Rohani & Griffiths, 1994). The amount of 10 mg of nisin was also dissolved in 1 ml of HCl (0.02 M, pH = 1.6) to prepare its stock solution. Different concentrations from this stock solution (200 to 3.125 µg/ml) were subsequently prepared (Dufour et al., 2003). The stock solutions of all the antimicrobial compounds were initially sterilized using a syringe filter (0.45 μm).

### 2.4 Determination of the MIC and MBC of the antimicrobials

The microdilution method was used to determine the minimal inhibitory concentration (MIC) of the antimicrobial compounds used in this study. First, 95 μl of BHI broth, 5 μl of the bacterial suspension (approximately 10^6^ CFU/ml), and 100 μl of different concentrations of the antimicrobial solution were dispensed in the wells of a 96‐well microplate. The microplate was vortexed (260 rpm (revolutions per minute), 30 s) and then incubated at 37°C for 24 h. After incubation, the bacterial growth was visually assessed and the lowest concentration of each antimicrobial in which bacterial growth (turbidity) was not observed, was recorded as the MIC (Mortazavi & Aliakbarlu, 2019). To determine the minimum bactericidal concentration (MBC), 5 μl from the wells with no visible growth (MIC and higher concentrations) was cultured on PCA plates and incubated at 37°C for 24 h. MBC was defined as the lowest concentration of an antimicrobial at which no bacterial colony was formed on PCA. Wells without bacterial suspension (100 μl broth + 100 μl antimicrobial) and wells without antimicrobials (195 μl broth + 5 μl of the bacterial suspension) were also designed as the sterility control and the growth control, respectively (Mortazavi & Aliakbarlu, 2019).

### 2.5 Determination of FIC (fractional inhibitory concentration)

The antibacterial interactions between pulegone/1,8‐cineole and monolaurin/nisin were examined by the FIC method. To determine the MIC individually, in the wells of the first row of a 96‐well microplate, 95 µl BHI broth, 5 µl bacterial suspension (≈10^6^ CFU/ml), and 100 µl of different concentrations of pulegone or 1,8‐cineole were added. Similarly, the wells of the first column of the microplate were filled with 95 µl BHI broth, 5 µl bacterial suspension, and 100 µl of different concentrations of monolaurin or nisin. In the remaining wells, 95 µl BHI broth, 5 µl bacterial suspension, and 50 µl from each antimicrobial were added. The combinations were as follows: 50 µl pulegone + 50 µl monolaurin, 50 µl pulegone + 50 µl nisin, 50 µl 1,8‐cineole + 50 µl monolaurin, and 50 µl 1,8‐cineole + 50 µl nisin. The final volume of each well was 200 µl (Nafis et al., 2019). The FIC index (FICi) was calculated according to the following equations:

$$\begin{array}{c} {\text{FIC}}_{X}\;\text{= MIC of compound X in combination with compound Y}/\\ \text{MIC of compound X alone} \end{array}$$

$$\begin{array}{c} {\text{FIC}}_{Y}\;\text{= MIC of compound Y in combination with compound X}/\\ \text{MIC of compound Y alone} \end{array}$$

$$\text{FICi}={\text{FIC}}_{X}+{\text{FIC}}_{Y}$$

The antibacterial interactions were interpreted as total synergistic (FICi ≤ 0.5), partial synergistic (0.5 < FICi ≤ 0.75), indifferent (0.75 < FICi ≤ 2), and antagonistic (FICi > 2) (Nafis et al., 2019).

### 2.6 Time‐kill method

To investigate the lethal effects of pulegone and 1,8‐cineole combined with nisin or monolaurin, 50 μl of bacterial suspension (≈10^8^ CFU/ml) was inoculated into tubes containing 4450 μl of BHI broth. Then, 500 µl from each antimicrobial solution was added to four tubes at the final concentration of MIC. In combined treatments, an amount of 250 µl of each antimicrobial at MIC concentration was also used as follows: 250 µl pulegone + 250 µl monolaurin, 250 µl pulegone + 250 µl nisin, 250 µl 1,8‐cineole + 250 µl monolaurin, and 250 µl 1,8‐cineole + 250 µl nisin. One tube was also considered as growth control, which contained BHI broth and bacterial culture. Subsequently, all of the tubes were incubated at 37°C. Serial dilutions from the tubes were prepared at 0, 2, 4, 8, and 24 h and cultured on the BHI agar. Finally, the colonies were enumerated after 24 h incubation at 37°C, and then the time‐kill curve was plotted (Avila et al., 1999; Lavigne et al., 2020).

### 2.7 Estimation of the cell constituents’ release

The effect of the antimicrobial compounds on the cell membrane integrity of *S. aureus* was evaluated by determining the release of cell constituents to the supernatant. The overnight culture of *S. aureus* in the BHI broth was centrifuged at 4000 ***g*** for 15 min, and the supernatant was discarded. The bacterial cells were resuspended in phosphate‐buffered saline (PBS) and centrifuged three times again at 4000 ***g*** for 15 min. Then, each individual antimicrobial compound (at 2 × MIC concentration) and its combination (at MIC concentration of each antimicrobial) were added to the bacterial suspension in PBS. A tube containing bacteria suspended in PBS was considered as a control. All tubes were incubated for 1 h in a shaker incubator at 250 rpm and 37°C. Then, the tubes were centrifuged at 13,400 ***g*** for 20 min. Eventually, the concentration of cell constituents in the supernatants was measured using a spectrophotometer at 260 nm. The PBS buffer was considered as a blank to zero the spectrophotometer (Mutlu‐Ingok & Karbancioglu‐Guler, 2017). To calculate cell constituent release, the optical density (OD) of the tube containing antimicrobial solution and the bacterial suspension was subtracted from that of the antimicrobial solution.

### 2.8 Statistical analysis

The experiments of MIC, MBC, and FIC were carried out three times, while time‐kill and cell constituents’ release experiments were conducted two times. The results of colony count were converted to log CFU/ml using Microsoft Excel 2013 and plotted using GraphPad Prism 9. The data were analyzed with SPSS 22.0 software (SPSS Inc., Chicago, LA) by analysis of variance (ANOVA) procedure, and Duncan test at 5% significant level.

## 3 RESULTS AND DISCUSSION

### 3.1 MIC and MBC of the antimicrobial compounds

The results of the MIC and MBC values of pulegone, 1,8‐cineole, monolaurin, and nisin against *S*. *aureus* are presented in Table 1. The MIC values of pulegone and 1,8‐cineole against *S*. *aureus* were 5.85 and 23.43 µl/ml, respectively. Meanwhile, the MBC values of these compounds were found to be 11.71 and 23.43 µl/ml, respectively. Therefore, pulegone was more active against *S. aureus* than 1,8‐cineole. Similar to our results, it was reported that the MIC value of 1,8‐cineole (eucalyptol) against *S. aureus* was 20 µl/ml (Zengin & Baysal, 2014). However, another study showed that MIC and MBC values of 1,8‐cineole against *S. aureus* were 1.25% and 5% (v/v), respectively (W. Wang et al., 2012). It has also been shown that the MIC values of pulegone and 1,8‐cineole against *S. aureus* were 1.8 and 3.6 mg/ml, respectively (Sonboli et al., 2006). Another work showed that the MIC value of 1,8‐cineole was 10 μl/mL against *S. aureus* (Honório et al., 2015). Meanwhile, the MIC value of pulegone was found to be 2.8 μl/ml against *S. aureus* (Amalich et al., 2016). According to the reported results, the MIC values could be varied. The antimicrobial performance of essential oils in vitro depends on various factors such as antimicrobial components, type of microorganism, culture medium, amount of inoculum, pH, temperature, and food composition (Tajkarimi et al., 2010; H. Zhou et al., 2014).

**TABLE 1:** The minimum inhibitory concentration (MIC) and minimum bactericidal concentration (MBC) values of pulegone, 1,8‐cineol (µl/ml), monolaurin (mg/ml), and nisin (µg/mL) against *Staphylococcus aureus*

Antimicrobial	MIC	MBC
Pulegone	5.85	11.71
1,8‐Cineol	23.43	23.43
Monolaurin	0.031	0.031
Nisin	6.25	6.25
Erythromycin^a^	8	8

^a^ µg/ml.

The MIC value of monolaurin was 0.031 mg/ml. This finding is similar to the results reported by other researchers (Raeisi et al., 2016). Evidence exists that monolaurin destroys the cell membrane by its lipophilic characteristics and inhibits the growth of Gram‐positive bacteria, while it has no effect on Gram‐negative bacteria (Delamare et al., 2007; Tajik et al., 2014). In one study, the MIC of monolaurin against *S*. *aureus* was 0.0625 mg/ml (Preuss et al., 2005), while Tangwatcharin and Khopaibool (2012) reported that the MIC value of monolaurin against *S. aureus* ATCC 25923 was 0.1 mg/ml. The other study demonstrated that monolaurin alone could inhibit *S. aureus* at a level of 128 μg/ml (pH = 7) and 16 μg/ml (pH = 5) but had no effect on *E. coli*. Therefore, the most potent antibacterial activity against *S. aureus* was observed at the lower pH (Aminzare et al., 2014). The MIC values of monolaurin against *S*. *aureus* ATCC 25923 and ATCC 1885 were 100 and 250 μg/ml, respectively (Sadiq et al., 2016; Tajik et al., 2014).

In this study, the MIC value of nisin was determined to be 6.25 µg/ml. In agreement with our finding, other researchers showed that the MIC value of nisin against *S. aureus* ranged from 2 to 32 µg/ml (Dosler et al., 2012). It was shown that the MIC value of nisin against *S. aureus* was 8 µg/ml (Zhao et al., 2014). However, another study showed that the MIC value of nisin against *S. aureus* strains was in the range of 16–32 µg/ml (Shi et al., 2017).

### 3.2 FIC

The results of FIC indices for the antimicrobial compounds (combination of pulegone with monolaurin/ nisin and 1,8‐cineole with monolaurin/nisin) are presented in Table 2. It was found that the combination of 1,8‐cineole and nisin had a synergistic effect against *S*. *aureus* with a FIC index of 0.5. Besides, the combination of pulegone and monolaurin resulted in a partial synergistic effect (FICi = 0.75). However, the combined use of 1,8‐cineole with monolaurin and pulegone with nisin was ineffective against *S. aureus*. Several studies have reported the antimicrobial interactions between nisin and other antimicrobials. For example, the combination of nisin and lactoperoxidase system showed a synergistic effect against *S*. *typhimurium* and *S*. *aureus* (Dufour et al., 2003). Furthermore, a synergistic effect between nisin and coenzyme Q has been reported against *S*. *aureus* isolates (Zhao et al., 2014). Another similar study showed a synergistic effect between nisin and cinnamaldehyde against *S*. *aureus* strains with the FIC value of 0.3 (Shi et al., 2017). It has been reported that the combined use of 1,8‐cineole and carvacrol could synergistically inhibit *Listeria monocytogenes, Aeromonas hydrophila,* and *Pseudomonas fluorescens* (De Sousa et al., 2012). In another study, synergy was found between 1,8‐cineole and aromadendrene against *S. aureus* (Mulyaningsih et al., 2010). Meanwhile, 1,8‐cineole in combination with limonene showed a synergistic effect against *S. aureus* (van Vuuren & Viljoen, 2007). However, it has been reported that the antibacterial effects of 1,8‐cineole in combination with α‐terpineol or linalool were additive (FICi = 1) against *S*. *aureus* (Zengin & Baysal, 2014).

**TABLE 2:** The fractional inhibitory concentration index (FICi) of the antimicrobials against *Staphylococcus aureus*

Antimicrobial combination	FIC	FICi	Function
Pulegone	0.25	0.75	Partial synergistic
Monolaurin	0.5		
Pulegone	1	1.5	Indifferent
Nisin	0.5		
1,8‐Cineol	1	1.122	Indifferent
Monolaurin	0.122		
1,8‐Cineol	0.25	0.5	Synergistic
Nisin	0.25		

### 3.3 Time‐kill assay

**FIGURE 1:**
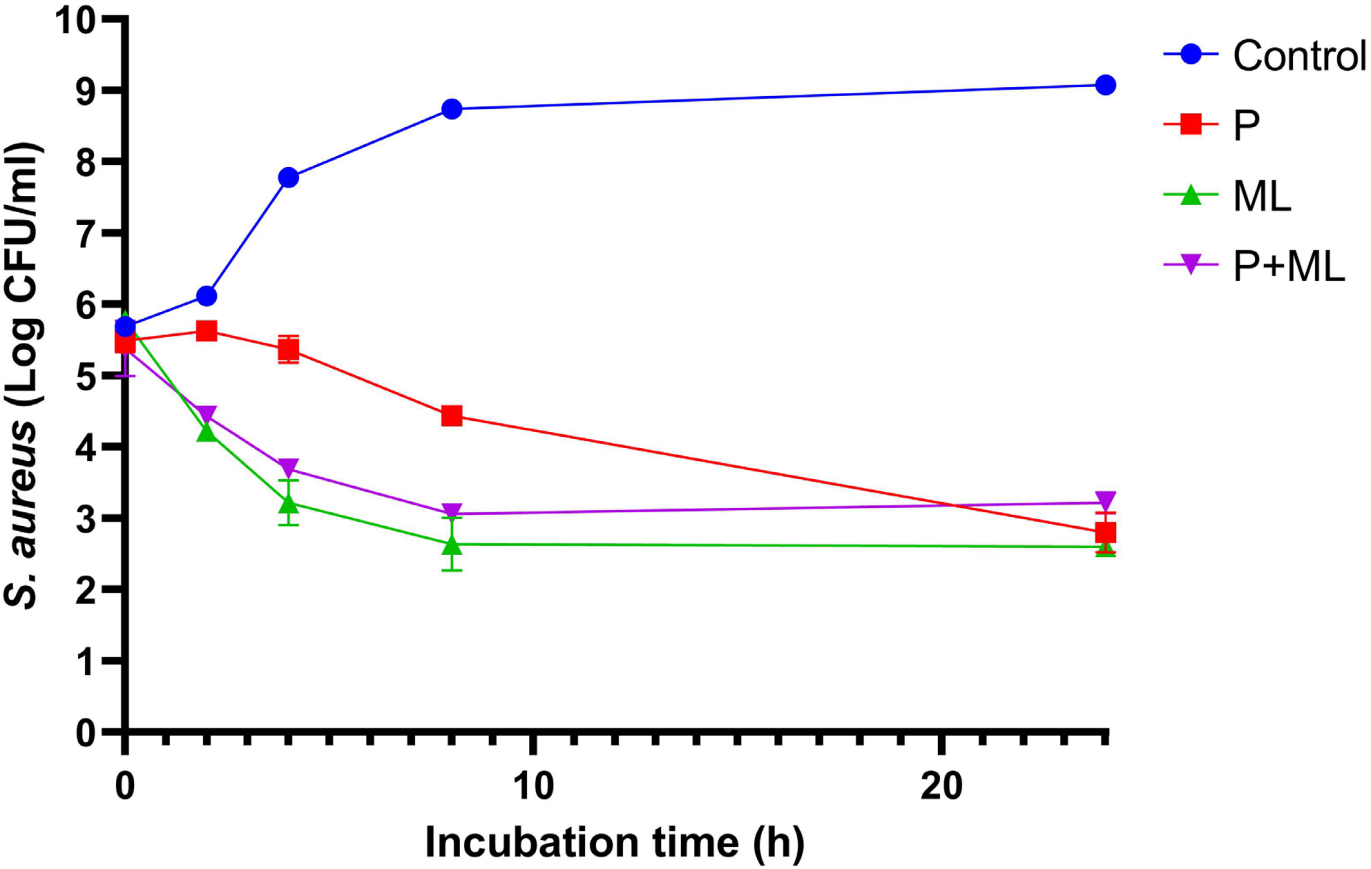
The antibacterial effects of pulegone, monolaurin, and their combination against *Staphylococcus aureus* (log CFU/ml) during 24 h incubation at 37°C. P: pulegone; ML: monolaurin

The antibacterial interaction between pulegone and monolaurin against *S*. *aureus* during 24 h incubation is shown in Figure 1. Within the first 8 h, monolaurin showed stronger antibacterial activity than pulegone against *S. aureus*. However, at the end of 24 h incubation, there was no significant difference between their effects. Within the first 2 h, no significant difference was detected between the antibacterial performance of monolaurin and the combination of pulegone and monolaurin (P+ML). Nevertheless, at subsequent incubation times, monolaurin had surprisingly stronger antibacterial activity than the combination of pulegone and monolaurin. The combination of pulegone and monolaurin had a significantly stronger antibacterial effect than pulegone alone at the first 8 h. After 24 h incubation, however, there was no significant difference between their effects. Furthermore, all treatments displayed significant effects on *S. aureus* at the end of incubation, and approximately a 6‐log reduction in bacteria counts was induced by the treatments compared to control.

**FIGURE 2:**
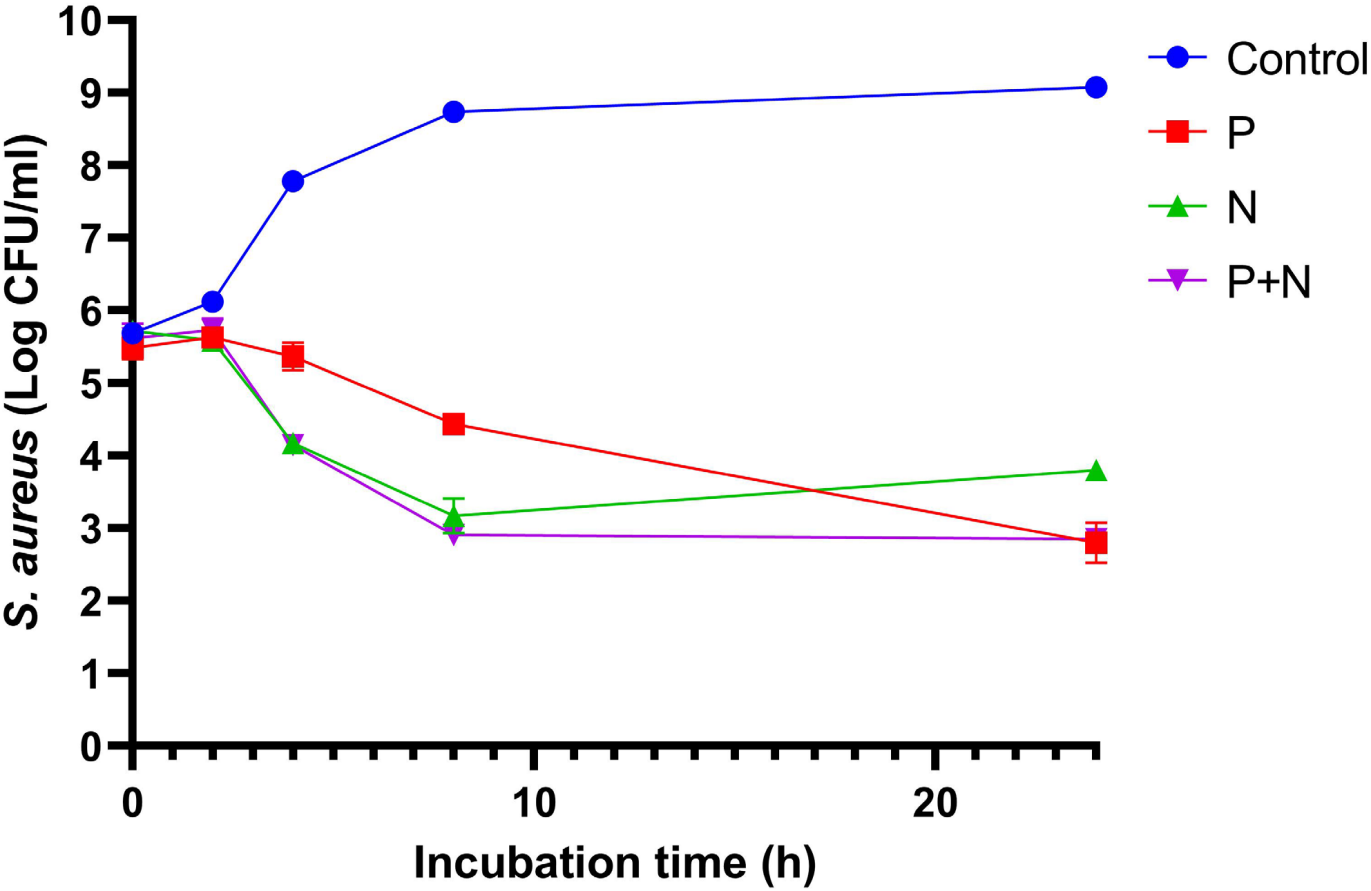
The antibacterial effects of pulegone, nisin, and their combination against *Staphylococcus aureus* (log CFU/ml) during 24 h incubation at 37°C. P: pulegone; N: nisin

Figure 2 shows the antibacterial performance of pulegone, nisin, and their combination. Within the first 8 h, the antibacterial activity of nisin and the combination of pulegone and nisin (P + N) were similar, and their effects were significantly stronger than pulegone alone. After 24 h, a weak regrowth was observed in nisin treatment.

**FIGURE 3:**
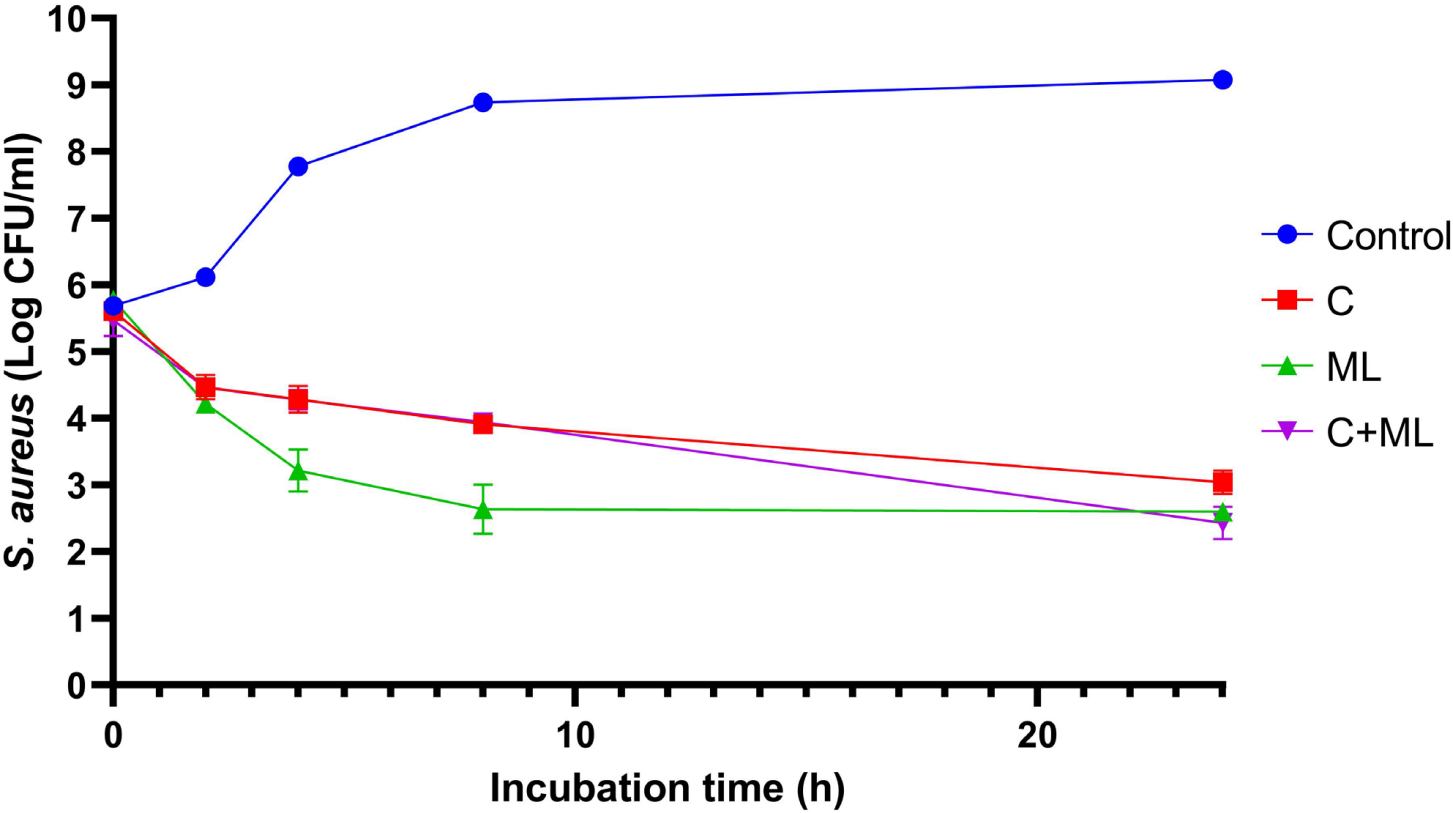
The antibacterial effects of 1,8‐cineol, monolaurin, and their combination against *Staphylococcus aureus* (log CFU/ml) during 24 h incubation at 37°C. C: 1,8‐cineol; ML: monolaurin

The antibacterial interaction between 1,8‐cineol and monolaurin is illustrated in Figure 3. Within the first 8 h, the antibacterial effect of 1,8‐cineol was equal to that of the combination of 1,8‐cineol and monolaurin (C+ML). However, monolaurin alone showed stronger antibacterial activity than other treatments. At the end of 24 h incubation, the antibacterial effect of monolaurin was similar to that of the combination of 1,8‐cineol and monolaurin, and the effects of these two treatments were stronger than that of 1,8‐cineol alone.

**FIGURE 4:**
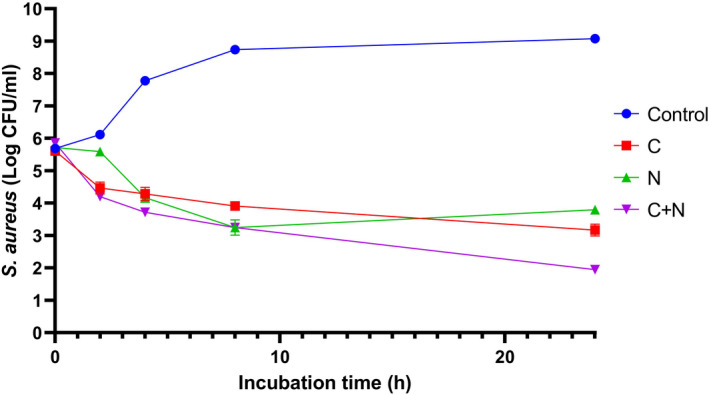
The antibacterial effects of 1,8cineol, nisin, and their combination against *Staphylococcus aureus* (log CFU/ml) during 24 h incubation at 37°C. C: 1,8‐cineol; N: Nisin

The antibacterial effect of 1,8‐cineole and nisin, individually and in combination, on *S. aureus* is depicted in Figure 4. During the first 4 h of incubation, the combination of 1,8‐cineole and nisin (C + N) showed significantly (*p* < .05) stronger antibacterial activity than the individual treatment of 1,8‐cineole or nisin. At the end of 24 h incubation, the combination of 1,8‐cineole and nisin also exhibited the greatest antibacterial performance against *S. aureus,* and more than 7‐log reduction in the bacterial count was induced by this treatment compared to control. Meanwhile, the antibacterial effect of 1,8‐cineole (5.9 log reduction) was higher than that of nisin (5.3 log reduction). Generally, the results of the time‐kill assay were in agreement with the FIC method.

Many studies have shown that some antimicrobials, such as nisin and monolaurin alone, are more effective against Gram‐positive bacteria but have no effect on Gram‐negative ones (Raeisi et al., 2016; Wang et al., 2020). However, it has been proven that the combined use of the antimicrobials can increase not only the antibacterial effect but also reduce the required dose of each antimicrobial (Wang et al., 2020). It has been reported that the combination of nisin and cell‐free supernatant of *Bacillus licheniformis* showed synergistic bactericidal activity against *S. aureus* after 4 h (He & Chen, 2006). The results of the time‐kill test in broth and pasteurized milk showed that nisin in combination with cinnamaldehyde had a synergistic antibacterial effect against *S. aureus* (Shi et al., 2017).

### 3.4 Cell constituents’ release

The cell membrane integrity was evaluated by measuring the release of cell constituents such as nucleic acids and proteins into the supernatant at 260 nm. The internal cell constituents were released into the supernatant following cell membrane disruption, and then the absorbance of the supernatant at 260 nm was increased. Table 3 shows the cell constituents’ release in *S. aureus* induced by individual antimicrobials and their combinations. It was found that the combination of 1,8‐cineole with nisin had the greatest effect on cell constituents’ release (OD = 1.231), followed by the combination of 1,8‐cineole with monolaurin (OD = 0.860). However, monolaurin alone showed the lowest effect on cell constituent's release. Compared to control, the combination of 1,8‐cineole with nisin and 1,8‐cineole with monolaurin increased the absorbance of the supernatant by 7.4 and 5.2 times, respectively.

**TABLE 3:** The cell constituents’ release in *Staphylococcus aureus* induced by individual (at 2 × MIC (minimum inhibitory concentration)) and combined antimicrobials (at MIC of each antimicrobial)

Antimicrobial treatments	OD_a_	OD_b_	OD_a_–OD_b_
Control	0.165 ± 0.014	–	0.165 ± 0.014
Pulegone	3≤	2.140	–
1,8‐Cineol	1.690 ± 0.018	1.100	0.590 ± 0.018
Monolaurin	0.368 ± 0.011	0.022	0.346 ± 0.011
Nisin	0.611 ± 0.031	0.156	0.455 ± 0.031
Pulegone + Monolaurin	3≤	1.938	–
Pulegone + Nisin	3≤	2.112	–
1,8‐Cineol + Monolaurin	1.710 ± 0.024	0.850	0.860 ± 0.024
1,8‐Cineol + Nisin	1.971 ± 0.016	0.740	1.231 ± 0.016

Note

OD_a_ = optical density of PBS + antimicrobial + bacterial suspension.

OD_b_ = optical density of PBS + antimicrobial.

A previous study indicated that the combination of α‐terpineol with eucalyptol (1,8‐cineole) caused the highest release of 260 nm absorbing materials from *S. aureus* (Zengin & Baysal, 2014). The results of another study showed that the combination of nisin and cinnamaldehyde induced more damage on the cell membrane of *S. aureus* compared to nisin or cinnamaldehyde alone (Shi et al., 2017). The combination of nisin and *Zataria multiflora* essential oil significantly increased the cell constituents’ release from *S. aureus* (Moosavy et al., 2008).

## 4 CONCLUSIONS

The present study investigated the antibacterial effects of pulegone and 1,8‐cineole alone and combined with monolaurin or nisin against *S. aureus*. The results showed that there was a synergistic effect between 1,8‐cineole and nisin against *S. aureus*. This combination also caused the highest release of cell constituents. Therefore, the combination of 1,8‐cineole and nisin could be considered as a novel and promising antibacterial combination. The combined use may reduce the required dose of each antibacterial compound and decrease the development of antibacterial resistance. However, further researches are required to evaluate the effectiveness of this combination in food models.

## CONFLICT OF INTEREST

The authors declare that they have no conflict of interest.

## Data Availability

The data that support the findings of this study are available on request from the corresponding author.
